# Household Knowledge of Clinical Risks, Storage, and Disposal of Leftover Antibiotics: A Multinational Study in Seven Developing Countries

**DOI:** 10.3390/antibiotics14121212

**Published:** 2025-12-02

**Authors:** Katia Iskandar, Reham Kotb, Michelle Cherfane, Joumana Yeretzian, Julia Bou Dib, Bahia Chahine, Souheil Hallit, Rohul Amin, Mohamed Bahlol, Feten Fekih-Romdhane, Faten Hamed, Mai Helmy, Mohammed Irfan, Jayaseelan Murugaiyan, Abdallah Y Naser, Esra’ O. Taybeh, Nebojša Pavlović, Deema Rahme, Marwan Akl, Pascale Salameh, Ana Tomas, Maarten Van Dongen

**Affiliations:** 1 Department of Biomedical Sciences, School of Pharmacy, Lebanese International University, Beirut P.O. Box 146404, Lebanon; 2 Department of Health and Social Work, School of Public Health, Lebanese University, Fanar P.O. Box 50501, Lebanon; 3 INSPECT-LB (Institut National de Santé Publique, d’Épidémiologie Clinique et de Toxicologie-Liban), Beirut P.O. Box 12109, Lebanon; 4 Department of Environmental and Public Health, College of Health Sciences, Abu Dhabi University, Abu Dhabi 59911, United Arab Emirates; 5 Department of Primary Health Care, High Institute of Public Health, Alexandria University, Alexandria 21526, Egypt; 6 Gilbert and Rose-Marie Chagoury School of Medicine, Lebanese American University, Byblos P.O. Box 36, Lebanon; 7 Center for Collaborative Research Initiatives in Public Health, Higher Institute of Public Health, Saint Joseph University of Beirut, Riad El Solh, Beirut P.O. Box 11-5076, Lebanonjulia.boudib1@usj.edu.lb (J.B.D.); 8 School of Medicine and Medical Sciences, Holy Spirit University of Kaslik, Jounieh P.O. Box 446, Lebanon; souheilhallit@hotmail.com; 9 Department of Psychology, College of Humanities, Effat University, Jeddah 21478, Saudi Arabia; 10 Applied Science Research Center, Applied Science Private University, Amman P.O. Box 541350, Jordan; 11 Bengal Overseas Ltd., Mohakhali, Dhaka 1212, Bangladesh; 12 Specialty of Pharmaceutical Management and Economics, Department of Pharmacy Practice and Clinical Pharmacy, Faculty of Pharmacy, Egyptian Russian University, Badr City 11829, Cairo Governorate, Egypt; ph_hossni@yahoo.com; 13 The Tunisian Center of Early Intervention in Psychosis, Department of Psychiatry “Ibn Omrane”, Razi Hospital, Manouba 2010, Tunisia; 14 Faculty of Medicine of Tunis, Tunis El Manar University, Tunis 2088, Tunisia; 15 Department of Psychology, College of Education, Sultan Qaboos University, Muscat P.O. Box 32, Oman; 16 Department of Restorative Dentistry, School of Dentistry, Federal University of Pelotas, Pelotas 96020-010, Brazil; irfan_dentart@yahoo.com; 17 Department of Biological Sciences, School of Engineering and Sciences (SEAS), SRM University-AP, Amaravati 522240, Andhra Pradesh, India; jayaseelan.m@srmap.edu.in; 18 Department of Applied Pharmaceutical Sciences and Clinical Pharmacy, Faculty of Pharmacy, Isra University, Amman P.O. Box 11622, Jordan; abdallah.naser@iu.edu.jo (A.Y.N.);; 19 Department of Pharmacy, Faculty of Medicine, University of Novi Sad, Hajduk Veljkova 3, 21000 Novi Sad, Serbia; nebojsa.pavlovic@mf.uns.ac.rs; 20 Department of Pharmacy Practice, Faculty of Pharmacy, Beirut Arab University, Beirut P.O. Box 11-5020, Lebanon; 21 Faculty of Pharmacy, Lebanese University, Hadath P.O. Box 6573/14, Lebanon; 22 Department of Primary Care and Population Health, University of Nicosia Medical School, 2417 Nicosia, Cyprus; 23 Department of Pharmacology, Toxicology, and Clinical Pharmacology, Faculty of Medicine, University of Novi Sad, Hajduk Veljkova 3, 21000 Novi Sad, Serbia; 24 AMR Insights, 1017 EG Amsterdam, The Netherlands

**Keywords:** self-medication, leftover antibiotics, household, developing countries, storage, disposal

## Abstract

Background: Self-medication with leftover antibiotics (SMLA) drives antimicrobial resistance (AMR), particularly in developing countries. This study examined knowledge–practice gaps regarding antibiotic use and handling among individuals with prior SMLA across seven developing countries. Methods: A cross-sectional study (February 2023–February 2024) included 3191 adults from Bangladesh, Brazil, Egypt, India, Jordan, Lebanon, and Serbia who reported previous leftover antibiotic use. The questionnaires assessed knowledge of antibiotic use (15 items), resistance (12 items), and SMLA risks (15 items). Storage and disposal practices were evaluated using dichotomized correct/incorrect measures. Results: Participants demonstrated above-average knowledge scores as follows: antibiotic use (54.4 ± 8.4), resistance (43.3 ± 6.1), and SMLA risks (58.4 ± 10.3). However, substantial practice gaps emerged. Only 21.9% properly disposed of leftover antibiotics, while 47.0% used household garbage. For storage, 55.1% used appropriate methods, but 32.6% stored antibiotics inappropriately, and 12.8% lacked protocols. Serbian participants showed the highest SMLA risk knowledge (64.3 ± 10.6), Bangladeshi participants the lowest (52.0 ± 8.5, *p* < 0.001). Women had superior knowledge (59.6 ± 10.4 versus 56.5 ± 9.8, *p* < 0.001) and storage practices (56.6% versus 52.7%, *p* = 0.031). Paradoxically, higher AMR knowledge was associated with poorer disposal practices (OR = 0.97, *p* < 0.001). Conclusions: Despite extensive theoretical knowledge, unsafe medication practices persist, revealing a critical knowledge–practice gap. Our findings challenge education-focused AMR approaches, suggesting cognitive awareness alone cannot drive behavioral change. Effective interventions must address structural barriers, cultural factors, and individual health beliefs beyond traditional knowledge-deficit models.

## 1. Introduction

Antimicrobial resistance (AMR) represents a pressing global health and development threat, disproportionately affecting developing countries [1]. In these nations, limited healthcare infrastructure, over-the-counter antibiotic availability, and loose regulatory oversight create ideal conditions for resistance emergence [1]. The impact of AMR is devastating clinically, environmentally, and economically [1,2]. An estimated 1.27 million deaths in 2019 are directly attributable to bacterial AMR, with Sub-Saharan Africa (24 deaths per 100,000) and South Asia (22 per 100,000) bearing the highest burden [2]. These estimates are projected to reach 1.91 million deaths globally by 2050 [2]. One of the most significant drivers of AMR is the inappropriate use of antimicrobial agents, particularly self-medication with leftover antibiotics (SMLA) [3,4,5]. This practice involves the consumption of unused portions of prescribed antimicrobials retained following treatment completion, with originating sources including pharmacies, online store purchases, or shared medications from family members and friends [3,4,5,6,7]. SMLA is particularly concerning in developing countries [4]. A systematic review and meta-analysis indicated that the prevalence of self-medication with antibiotics in the Middle East is 38.8%, with 50% attributable to household leftovers [5]. SMLA drivers are complex, significantly contributing to the widespread use of this practice [8,9]. The clinical and environmental risks associated with this SMLA are particularly detrimental. Inappropriate use of leftover antibiotics (LAs) increases the likelihood of adverse drug reactions, inadequate medication selection, incomplete treatment courses, prolonged recovery periods, and elevated risk of medical complications [3,4,5,6,7]. SMLA can also create ideal conditions for resistance development through subtherapeutic dosing, inappropriate selection, and increased selective pressure on bacterial populations [10,11,12]. The environmental impact stems from improper storage and disposal practices [10,11,12], with pharmaceutical compounds entering waterways and soil through household waste streams [13,14]. These residues create selection pressure on environmental microbiota, facilitating horizontal gene transfer of resistance determinants that eventually cycle back to clinical settings, creating a dangerous feedback loop [13,14].

In developing countries, knowledge about SMLA is shaped by socioeconomic and cultural determinants [3,4,5,6,7]. Limited healthcare accessibility and financial constraints transform self-medication from choice to a perceived necessity [3,4,5,6,10,11,12], while cultural norms promoting familial medicine sharing normalize practices that contribute to suboptimal antibiotic utilization [3,4,5,6,7]. A clear knowledge–attitude–practice (KAP) gap exists: while participants from low- and middle-income countries (LMICs) often demonstrate better theoretical AMR awareness than those from high-income countries (HICs), they exhibit poorer attitudes toward antimicrobial use in practice [15]. The Health Belief Model (HBM) provides further insights into this discrepancy, suggesting that antibiotic behaviors are influenced not only by knowledge but also by perceived susceptibility to complications, severity of untreated infections, perceived benefits of self-medication, and perceived barriers to accessing healthcare [16]. Research indicates that public knowledge about appropriate antibiotic use remains deficient globally [15,16,17,18,19], with the World Health Organization (WHO) 2015 survey revealing persistent misconceptions about antibiotic efficacy and resistance mechanisms [17]. Paradoxically, higher educational levels sometimes correlate with increased SMLA, possibly because educated individuals overestimate their self-diagnostic abilities [6,20].

Investigating the knowledge and practice gaps about SMLA, one of the predominant drivers of AMR in developing countries [1], is crucial for informing public health policies, targeted educational interventions, and effective antimicrobial stewardship programs. In the Middle East, pharmacy students have been found to have widespread gaps in antibiotic stewardship. Additionally, there is evidence that cost-related nonadherence contributes to improper medication use and storage, which may exacerbate unsafe practices surrounding LA use in household settings [21,22,23]. This study focuses on seven developing countries across four continents (Bangladesh, Brazil, Egypt, India, Jordan, Lebanon, and Serbia), representing diverse healthcare systems, regulatory environments, and socioeconomic contexts in regions where SMLA prevalence is substantial [5]. The aim is to examine the knowledge of antibiotics, resistance, and risks associated with SMLA, storage, and disposal practices among individuals with prior use across seven developing countries. The findings can provide actionable insights to address this critical dimension of one of the top ten global public health threat facing humanity [24].

## 2. Results

### 2.1. Sociodemographic Factors

The study included a total of 3191 participants from seven developing countries. Table 1 below shows the distribution of participants according to their country of residence. Table 1 also describes the sociodemographic distribution of the participants. The results showed that the participants’ mean age was 29.6 years old, and 82.1% attended university. In addition, around two-thirds of the participants were female (61.5%), single (63.5%), not healthcare providers (68.2%), and living in crowded houses (62.7%).

### 2.2. Knowledge of Antibiotics, Uses, Resistance, and SMLA-Associated Risks

Table 2 indicated that participants demonstrated above-average scores on all three scales assessing the following knowledge: knowledge of antibiotics (AB) and their use (mean score = 54.5 ± 8.4 out of 75), knowledge of antibiotic resistance (mean score = 43.3 ± 6.1 out of 60), and knowledge about clinical risks associated with SMLA (mean score = 58.4 ± 10.3 out of 75).

**Figure 1:**
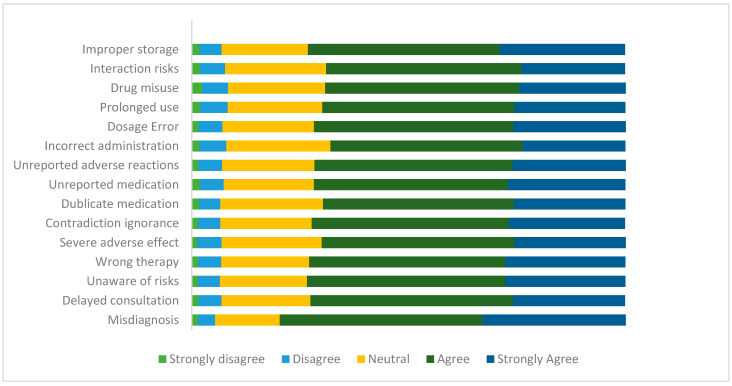
Participants’ knowledge of the risks associated with using LAs in percentage (n = 3191).

Figure 1 below shows in more detail the participants’ distribution regarding their knowledge about the risk of using LAs. Overall, the results indicate that participants have a moderate level of awareness regarding these risks.

Table 3 showed a significant association between the knowledge about clinical risks associated with SMLA and several sociodemographic variables, including country (*p* < 0.001), gender (*p* < 0.001), marital status (*p* < 0.001), education level (*p* < 0.001), healthcare provider status (*p* < 0.001), and age (*p* < 0.001). Serbian participants scored the highest (64.3 ± 10.6), while Bangladeshi participants scored the lowest (52.0 ± 8.5). Women, single individuals, healthcare providers, and those with higher education exhibited superior knowledge compared to their counterparts, whereas age showed a weak negative correlation (r = −0.09) with the knowledge scale.

Additionally, knowledge of SMLA risks was positively and significantly correlated with both knowledge of AB and their use, as well as knowledge of AMR (*p*-value < 0.001, for each scale). These correlations were of moderate strength.

Table 4 identified significant determinants of knowledge of SMLA risks. Regarding country of residence, participants from Jordan (B = −2.16, *p* < 0.001) and Bangladesh (B = −3.64, *p* < 0.001) demonstrated significantly lower knowledge compared to Egyptians, while Serbian (B = 1.72, *p* = 0.007, *p* < 0.001) participants showed significantly higher knowledge. Men demonstrated lower knowledge than women (B = −1.13, *p* < 0.001), and participants with moderate education levels showed lower knowledge of SMLA risks than those with higher education (B = −1.10, *p* = 0.016). Greater knowledge in areas including antibiotics and their use (B = 0.34, *p* < 0.001) and antibiotic resistance (B = 0.45, *p* < 0.001) were all significantly associated with higher knowledge scores regarding SMLA clinical risks.

### 2.3. Storage and Disposal Methods of LAs

Regarding LAs storage, results revealed that 43.1% and 42.4% of participants lock their antibiotics in a cabinet out of children’s reach and keep them in a refrigerator, respectively. However, 32.6% of participants store their LAs in a drawer or closet, while 12.8% have no idea where to store the LAs.

The participants reported using various methods to discard leftover or expired antibiotics. Although the optimal method for handling LAs is to return them to a health facility, pharmacy, or manufacturing company, only 21.9% of participants adhered to this method, while nearly half (47.0%) threw their LAs in their household garbage.

Table 5 identified significant associations between appropriate antibiotic-handling practices and several factors. Regarding storage practices, significant associations were observed with country of residence (*p* < 0.001), with Lebanese, Egyptian, and Indian participants demonstrating the highest adherence (58.4%, 58.3%, and 58.0%, respectively), while Bangladeshi participants showed the lowest (36.0%). Women were more likely to store antibiotics correctly (56.6% vs. 52.7%, *p* = 0.031), and higher-educated participants showed better adherence (55.6%) compared to the less educated (vs. 36.5%, *p* = 0.011). Incorrect storage practices were significantly associated with knowledge about antibiotics and antibiotic use (*p* < 0.001).

For disposal practices, significant associations were found with country of residence (*p* < 0.001), with Serbian participants showing the highest adherence (60.8%) and Jordanian participants the lowest (7.1%). A significant relationship was observed with the crowding index, as participants from uncrowded households were more likely to adopt correct practices (26.1% vs. 19.4%, *p* < 0.001), and those with healthcare provider status showed better adherence (28.7% vs. 18.7%, *p* < 0.001). Higher knowledge regarding antibiotics and their use, as well as antibiotic resistance, were associated with correct disposal practices (*p* < 0.001 for both scales).

The binary logistic analyses identified significant determinants of antibiotic-handling practices (Table 6). For storage practices, Brazilian participants were 2.17 times less likely to adopt proper storage techniques compared to Egyptians (OR = 0.46, *p* < 0.001). In addition, participants with greater knowledge of antimicrobial resistance appeared more likely to store antibiotics correctly (OR = 1.02, *p* = 0.025). Counterintuitively, those with fewer misconceptions about antibiotics appeared less likely to store antibiotics correctly (OR = 0.96, *p* < 0.001).

For disposal practices, Serbian participants were 3.19 times more likely to follow correct practices compared to Egyptians (*p* < 0.001), while Bangladeshi participants were 5.6 times less likely (OR = 0.18, *p* < 0.001). Participants who are healthcare providers were more likely to adopt correct disposal practices (OR = 1.85, *p* < 0.001) compared to those who were not. Paradoxically, greater knowledge of antibiotic resistance was associated with a lower likelihood of correct disposal (OR = 0.97, *p* < 0.001).

## 3. Discussion

This study included 3191 participants across seven developing countries from four continents, revealing a significant knowledge–practice gap of antibiotics and their actual practices regarding SMLA. While participants demonstrated above-average knowledge scores across all domains, including antibiotic use (54.4 ± 8.4 out of 75), resistance (43.3 ± 6.1 out of 60), and SMLA risks (58.4 ± 10.3 out of 75), their practices were concerning. All participants reported SMLA engagement; however, only 21.9% properly disposed of LAs, and around 40% used appropriate storage methods. These findings suggest a critical gap between theoretical knowledge and practical implementation, indicating that cognitive awareness alone may be insufficient to drive appropriate antibiotic use behaviors.

Through the HBM lens [25,26], the results imply that despite adequate knowledge scores (mean SMLA risk knowledge: 58.4 ± 10.3 out of 75), participants’ poor practices indicate low perceived susceptibility to AMR consequences. Cognitive awareness of risks appears insufficient to change behavior when convenience factors and economic considerations serve as perceived benefits of SMLA that outweigh known risks [26]. Low adherence to proper disposal protocols (21.9%) suggests that practical barriers, such as a lack of accessible disposal facilities, compound the knowledge–practice gap. The persistent SMLA engagement despite risk awareness aligns with HBM constructs of perceived threat versus self-efficacy [25,26]. This theoretical framework helps explain our counterintuitive finding that better AMR knowledge predicted poorer disposal practices (OR = 0.97, *p* < 0.001). These results suggest that when individuals perceive systemic barriers as insurmountable, increased knowledge may paradoxically reinforce fatalistic behaviors. In practice, studies have demonstrated a complex relationship where increased awareness of AMR does not always translate into better behaviors and may even be associated with poorer disposal practices [27,28,29].

However, the interpretation of these findings requires validation through longitudinal research to confirm causal mechanisms underlying the paradoxical knowledge and behavior association.

Geographical variations reveal structural determinants of medication behaviors that transcend individual knowledge. Serbia’s superior performance (60.8% proper disposal; SMLA risk knowledge 64.3 ± 10.6) likely reflects its mandatory take-back programs under EU accession pharmaceutical reforms [30,31]. However, a comprehensive review of 48 international studies [32] demonstrated that “systems are not sufficiently effective”, even in countries with established take-back programs, highlighting the persistent gap between policy implementation and behavioral outcomes, consistent with our data [32]. Conversely, Jordan’s concerning disposal results (7.1% proper disposal), despite moderate SMLA risk knowledge (54.6 ± 9.0), exemplify how fragile health systems and the cultural normalization of medication sharing [33] can override individual knowledge. These cross-national disparities align with Rogowska and Zimmermann’s (2022) [32] conclusion that multiple factors beyond legislation, including public awareness, infrastructure accessibility, and cultural attitudes, determine disposal practices. Our findings substantiate the Theory of Planned Behavior’s emphasis on subjective norms and control beliefs [34] and Rogowska and Zimmermann’s ecological framework, demonstrating that national policy environments create behavioral opportunity structures that mediate knowledge translation into practice. These theories help explain our counterintuitive finding that higher AMR knowledge predicted poorer disposal practices (OR = 0.97, *p* < 0.001), as Rogowska and Zimmermann [32] identified that specific education about environmental impacts and proper disposal protocols, rather than general antibiotic knowledge.

Beyond individual behavioral factors, these knowledge–practice gaps may reflect systemic epistemic injustice in antimicrobial resistance governance [35,36,37]. Epistemic injustice, the systematic marginalization of knowledge systems [37], helps explain why communities may resist biomedical AMR narratives. Fricker (2007) [37] describes two key forms: (1) testimonial injustice, when local health epistemologies (e.g., antibiotic reuse as an adaptation to healthcare shortages) are excluded by biomedical frameworks; and (2) interpretive injustice, when communities lack shared concepts to articulate medication experiences within global AMR discourse.

Our findings demonstrate this dynamic through several key results. The dismissal of AMR as a ‘non-local’ problem (67.5%) likely represents not ignorance but resistance to global narratives that privilege abstract resistance risks over immediate survival needs. Similarly, so-called ‘misconceptions’ about antibiotics for viral infections (22%) may reflect rational adaptations in contexts where biomedical care is inaccessible, a clear case of testimonial injustice, where community expertise is marginalized. These patterns align with growing critiques of deficit-based AMR education [38], interpreting non-compliance as ignorance rather than investigating structural drivers.

The consistent gender pattern in medication management—wherein women demonstrate superior SMLA risk knowledge (59.6 ± 10.4 vs. 56.5 ± 9.8, *p* < 0.001) and better storage practices (56.6% vs. 52.7%, *p* = 0.031)—aligns with gender role theory [39,40,41], which suggests that socially constructed caregiving responsibilities enhance both health knowledge acquisition and the implementation of protective behaviors. This pattern reflects the gendered nature of health work, where women’s traditional roles as family health managers translate into more comprehensive medication stewardship practices.

Similarly, the education paradox manifests when moderately educated groups demonstrate suboptimal storage practices (OR = 0.56). These observations support the theoretical framework proposed by Horne (2013) [42], which conceptualizes health-related behaviors reflecting individuals’ assessments of perceived needs weighed against potential concerns.

This study identifies three critical practice gaps requiring urgent intervention: unsafe medication storage (32.6% using inappropriate locations, 12.8% lacking storage awareness), improper disposal methods (47% using household waste, contributing to environmental AMR reservoirs), and a significant knowledge–practice decoupling (despite high knowledge scores of 8.2 ± 1.6/10). These findings necessitate paradigm shifts from awareness-raising to behavior-informed interventions, from global AMR narratives to localized consequence framing, and knowledge transmission to structural enablers such as accessible disposal systems. The results demonstrate that closing the knowledge–practice gap requires moving beyond cognitive deficit models to address multi-level determinants of medication behaviors. Effective antimicrobial stewardship must integrate policy-level pharmaceutical governance, community-appropriate behavioral nudges [43,44], gender-sensitive practice supports, and ecological approaches to medication lifecycle management to successfully bridge the chasm between antibiotic knowledge and responsible use practices in developing countries contexts.

### Limitations

Several limitations warrant consideration when interpreting these findings:

- The reliance on self-reported behaviors introduces potential social desirability bias, leading to the underestimation of inappropriate practices.
- The cross-sectional design precludes establishing causal relationships between knowledge and practices, limiting our ability to determine whether knowledge improvements precede behavioral changes.
- The sample’s demographic composition, predominantly consisting of young, highly educated females, limits the generalizability of the findings, particularly to lower-educated and rural groups, and may overestimate general awareness while underestimating unsafe practices.
- While the study employed validated knowledge assessment tools, the behavioral measures did not capture the frequency, duration, or contextual triggers of self-medication practices, which would offer more nuanced intervention targets.
- The multinational sampling approach may not reflect country-specific determinants that require tailored interventions. Country-stratified analyses with sufficient statistical power would enable more contextually appropriate recommendations while maintaining cross-national comparative insights.

Despite these limitations, the study’s large sample size, multinational scope, and consistent measurement protocols provide robust evidence of the knowledge–practice gap that can inform policy and future research directions in antimicrobial stewardship.

## 4. Materials and Methods

### 4.1. Study Design and Participants

A cross-sectional study was conducted from February 2023 to February 2024 across a multinational research network. The study included seven developing countries, including Egypt (n = 1505), Lebanon (n = 488), Jordan (n = 421), Serbia (n = 240), Bangladesh (n = 236), Brazil (n = 182), and India (n = 119). Eligible participants were adults aged 18 years or older residing in the participating countries who reported using LAs.

### 4.2. Measurements

#### 4.2.1. Sociodemographic Characteristics

Sociodemographic characteristics collected in this study included age, gender, marital status, country of residence, educational level, being a healthcare professional, and the household crowding index (HHCI), a measure used to assess residential overcrowding, calculated by dividing the number of people living in the house by the total rooms excluding kitchens and bathrooms [45]. Participants were then classified into two groups, those living in non-crowded households (HHCI < 1) and those living in crowded households (HHCI ≥ 1).

#### 4.2.2. Knowledge About Antibiotic Use, Resistance, and Risks Associated with SMLA

Knowledge of antibiotic use focused on antibiotic effects, use, and side effects, and included 15 statements adapted from previous studies [46,47]. Knowledge of AMR comprised 12 statements adapted from previous research [48,49]. It focused on AMR spread mechanisms, global impact, personal health implications, transmission patterns between humans and animals, and contributing factors to resistance development. Knowledge of risks associated with SMLA use included 15 statements adapted from previous studies [50,51]. It assessed the knowledge about the risks and potential dangers associated with SMLA, including understanding inappropriate drug use, safety concerns, diagnostic errors, adverse effects, therapeutic failures, and economic consequences.

For each knowledge item, respondents rated their agreement on a 5-point Likert scale ranging from “strongly disagree” to “strongly agree.” Reverse coding was applied where necessary for negatively phrased statements. A higher score indicated better knowledge about self-diagnosis and treatment decision risks.

#### 4.2.3. Storage and Disposal of Leftover Antibiotics

Storage of LAs was assessed using the question “Where do you keep your antibiotic leftovers?” with the following four response options: in the refrigerator, in a locked cabinet out of children’s reach, in a drawer/closet, and “do not know” [52,53,54]. The variable was dichotomized into correct (keeping antibiotics either in the refrigerator or in a locked cabinet) vs. incorrect practices.

Disposal of LAs was determined through the question “What do you do with leftover antibiotics?” Response options are as follows: returning them to a health facility/pharmacy/company, throwing them in household garbage, throwing them into the sink, bringing them to the company’s drug box, flushing them down the toilet, giving them to friends or relatives, and burning them [52,53,54]. This variable was also dichotomized into correct (return them to a health facility, pharmacy, or manufacturing company) vs. incorrect disposal practices.

### 4.3. Data Collection

Google Forms, a cloud-based survey powered by Google, was used to create the online survey. Data was collected using volunteer sampling through university email platforms in all participating countries. To increase the response rate, snowball sampling techniques [30] were implemented by asking university students to share the link of the online survey with their families and social networks via various social media platforms (WhatsApp, Facebook, Instagram, and LinkedIn, current versions available during the data collection period of February 2023 to February 2024 on iOS and Android devices). The procedure varied slightly in each country, contributing to different participation rates.

The questionnaire was translated from English into Arabic, Serbian, and Portuguese following WHO translation guidelines [55]. The translated version was then back-translated to English again. The English versions were compared, with minor discrepancies corrected by consensus between the translators and the principal investigator in each country. The questionnaire (Appendix A) was pilot-tested before distribution to check for clarity and acceptability of the questions.

### 4.4. Statistical Analysis

Data management and analysis were conducted using SPSS 27. Sociodemographic variables, as well as storage and disposal of LAs, were described using frequencies and percentages, while all knowledge scores were described using means and standard deviations. Given the large sample size with skewness and kurtosis values within acceptable ranges (−0.382 and 0.387, respectively, for knowledge about clinical risks associated with SMLA), parametric tests were considered robust and were applied.

The knowledge about clinical risks associated with SMLA scale was validated in the Egyptian sample (n = 1505) through Exploratory Factor Analysis (EFA) using the principal component method and varimax rotation. EFA was carried out to extract items into factors and thus examine the validity of the knowledge construct of the questionnaire. One potential factor was identified, with a total of 15 items, which explained 57.5% of the variance. The factor loadings varied from 0.643 to 0.813. The overall reliability of the questionnaire was evaluated by calculating Pearson’s correlation coefficient and Cronbach’s alpha index. The questionnaire showed high internal reliability with a Cronbach’s alpha value of 0.947. The correlation matrix was used to assess the degree of correlation between all pairs of items on a questionnaire. None of the correlations exceeded 0.75, indicating the absence of item redundancy. Moreover, item–total correlations varied between 0.60 and 0.78, indicating a strong correlation between each item and the total score (excluding the item itself).

Independent samples *t*-test and ANOVA were performed to compare means between knowledge about the risks associated with SMLA and the sociodemographic and knowledge about antibiotic transmission variables. Pearson correlations were used to test the strength and direction of the linear relationship between all other knowledge scores. Multivariable linear regression models were presented to identify the predictors of risks associated with SMLA. The models included variables that demonstrated significant associations in the bivariate analysis.

Chi-square tests were performed to test statistical difference between storage and disposal of LAs and the sociodemographic and knowledge about antibiotic transmission variables. Independent samples *t*-tests were used to compare means with other knowledge scores. Binary logistic regression models, including all significant variables from the bivariate analysis, were applied to explore the factors associated with the correct antibiotic storage and disposal methods. A *p*-value of 0.05 was considered significant at all levels.

### 4.5. Sample Size Calculation

The minimum sample size was calculated using the G-Power software, version 3.0.10. Taking into consideration that a stratified analysis per country applies, the minimum sample size calculation was determined as follows: the calculated effect size was 0.0526, related to the omnibus test of multiple regression. The minimum sample required was n  =  120 per country, considering an alpha error of 5%, a power of 80%, and allowing 20 predictors in the model.

### 4.6. Ethical Approval

The Institutional Review Board at the Lebanese International University (LIU) approved this study under code number 2021RC-013-LIUSOP on 16 May 2021. The study complies with the Declaration of Helsinki [56]. The participating universities granted permission for the involvement of their students. Before filling out the online survey, participants were briefed about the study objectives and their right to withdraw at any time. Informed consent was obtained from all participants by including a consent statement at the beginning of the survey. Participants were required to read the statement and click a checkbox indicating their agreement to participate to proceed with the survey. Participants did not receive any financial reward for their participation. The online survey was anonymous and voluntary. Collected data were encrypted, stored in password-protected computers, and presented as de-identified electronic files in Microsoft Excel and SPSS.

## 5. Conclusions

This multinational investigation reveals a critical paradox in AMR prevention: extensive theoretical knowledge of antibiotic use, resistance, and risks of SMLA parallels persistent unsafe medication practices. Our findings fundamentally challenge education-centric approaches to AMR mitigation by demonstrating that cognitive awareness alone cannot drive behavioral change in antibiotic management.

The documented knowledge–practice gap reflects complex interactions between individual health beliefs, structural barriers, and cultural epistemologies rather than simple educational deficits. Notably, higher AMR knowledge was associated with poorer disposal practices, suggesting that awareness without accessible alternatives may foster fatalistic behaviors among communities facing systemic constraints.

Effective AMR interventions must transcend traditional deficit-model approaches to address the multifaceted drivers of antibiotic misuse. This requires (1) multi-level coordination, (2) structural interventions, including enhanced disposal infrastructure and context-specific regulatory frameworks, (3) gender-responsive strategies leveraging women’s demonstrated knowledge and stewardship capabilities, and (4) culturally competent approaches that integrate local health epistemologies with biomedical frameworks.

Our results directly advance multiple Sustainable Development Goals, positioning AMR as a cross-cutting challenge linking health equity (SDG 3), environmental sustainability (SDGs 6, 12), gender equality (SDG 5), and social justice (SDG 10). This interconnectedness demands integrated policy responses that bridge health, environmental, and equity agendas.

Future investigations should employ longitudinal mixed-methods designs to establish causal pathways between knowledge and practice, explore cultural frameworks of medication use, and evaluate the comparative effectiveness of structural versus educational interventions through implementation of science approaches.

This study contributes to the growing recognition that AMR is not merely a biomedical problem but a complex socioecological challenge. Moving beyond simplistic knowledge-deficit models, there is a need for an ecological approach to antimicrobial stewardship that acknowledges medication management as a sociotechnical system embedded within broader patterns of inequality and resource distribution. This comprehensive framework can effectively contribute to the fight against the growing threat of AMR in resource-constrained settings.

## Figures and Tables

**Table 1:** Sociodemographic distribution of participants (n = 3191).

Characteristic	n	%	
Country			
Egypt	1505	47.2	
Lebanon	488	15.3	
Jordan	421	13.2	
Serbia	240	7.5	
Bangladesh	236	7.4	
Brazil	182	5.7	
Gender			
Female	1963	61.5	
Male	1228	38.5	
Marital Status			
Single	2027	63.5	
Married	1083	33.9	
Divorced	58	1.8	
Widow	23	0.7	
Education			
Uneducated	16	0.5	
Primary School	47	1.5	
Middle School	91	2.9	
High School	416	13	
University	2621	82.1	
Healthcare provider			
No	2177	68.2	
Yes	1014	31.8	
HHCI ^1^			
<1 (no crowding)	1190	37.3	
≥1 (crowding)	2001	62.7	
	Mean ± S.D. ^2^	Minimum	Maximum
Age (years)	29.3 ± 10.6	18	98

^1^ HHCI: household crowding index, it measures the relationship between the number of occupants and the dwelling space available, a household is considered crowded if HHCI ≥ 1. ^2^ S.D.: standard deviation.

**Table 2:** Knowledge about antibiotics use, resistance, and the risks associated with SMLA (n = 3191).

Knowledge Scales	Mean ± S.D.	Minimum	Maximum
Knowledge of AB and AB use ^1^	54.4 ± 8.4	17	75
Knowledge about AB resistance ^2^	43.3 ± 6.1	20	60
Knowledge about risks associated with SMLA ^3^	58.4 ± 10.3	15	75

AB: antibiotics; S.D.: standard deviation. ^1^ this scale is derived from 15 items assessing participant’s knowledge of AB and their use, with total scores ranging from 15 to 75 (Cronbach alpha = 0.839). A higher score indicates better knowledge. ^2^ this scale is derived from 12 items assessing participant’s knowledge of AB resistance, with total scores ranging from 12 to 60 (Cronbach alpha = 0.749). A higher score indicates better knowledge. ^3^ this scale is derived from 15 items assessing participant’s knowledge of clinical risks associated with SMLA, with total scores ranging from 15 to 75 (Cronbach alpha = 0.942). A higher score indicates better knowledge.

**Table 3:** Association between knowledge of the risks of SMLA and independent variables (n = 3191).

Knowledge About Clinical Risks Associated with SMLA				
Variable	Mean ± S.D.	95% IC	Test	*p*-Value
Country			43.5	<0.001
Egypt (n = 1505)	59.3 ± 10.2	58.8–59.8		
Lebanon (n = 488)	58.6 ± 10.0	57.8–59.6		
Jordan (n = 421)	54.6 ± 9.0	53.8–55.5		
Serbia (n = 240)	64.3 ± 10.6	63.0–65.7		
Bangladesh (n = 236)	52.0 ± 8.5	50.9–53.1		
Brazil (n = 182)	59.9 ± 9.3	58.5–61.2		
India (n = 119)	58.1 ± 11.2	56.0–60.1		
Gender			8.38	<0.001
Female (n = 1963)	59.6 ± 10.4	59.2–60.1		
Male (n = 1229)	56.5 ± 9.8	55.9–57.1		
Marital status			5.77	<0.001
Single (n = 2027)	59.2 ± 10.2	58.7–59.6		
Ever married (n = 1164)	57.1 ± 10.5	56.5–57.7		
Education level			27.23	<0.001
Low or no education (n = 63)	52.8 ± 9.7	50.4–55.3		
Moderate education (n = 507)	56.1 ± 10.5	55.1–57.0		
Higher education (n = 2621)	59.0 ± 10.2	58.6–59.4		
Crowding index			−0.25	0.802
No crowding (n = 1190)	58.4 ± 10.5	57.8–59.0		
Crowding (n = 2001)	58.5 ± 10.2	58.0–58.9		
Healthcare provider			−8.79	<0.001
No (n = 2177)	57.3 ± 10.2	56.9–57.8		
Yes (n = 1014)	60.7 ± 10.2	60.1–61.4		
	Pearson’s correlation coefficient		*p*-value	
Age	−0.09		<0.001	
Knowledge of AB and AB use	0.47		<0.001	
Knowledge of AB resistance	0.46		<0.001	

IC: 95% confidence interval of the mean; S.D.: standard deviation; AB: antibiotics.

**Table 4:** Multivariate linear regression analysis of factors associated with the knowledge of risks of SMLA (n = 3191).

	LA Administration Risks			
	Unstandardized B	Standardized Beta	95% CI	*p*-Value
Country Egypt (reference)				
Lebanon	0.06	0	−0.84, 0.95	0.904
Jordan	−2.16	−0.07	−3.12, −1.24	<0.001
Serbia	1.72	0.04	0.47, 2.97	0.007
Bangladesh	−3.64	−0.09	−4.92, −2.37	<0.001
Brazil	0.42	0.01	−0.96; 1.80	0.553
Gender Female (reference)				
Male	−1.13	−0.05	−1.77, −0.48	<0.001
Marital status Single (reference)				
Ever married	−0.44	−0.02	−1.30, 0.43	0.323
Education level High (reference)				
Low education	−0.46	−0.01	−2.73, 1.80	0.689
Moderate education	−1.1	−0.04	−2.00, −0.20	0.016
Healthcare provider No (reference)				
Yes	−0.33	−0.02	−1.04, 0.39	0.372
Age	−0.02	−0.02	−0.06, 0.02	0.381
Knowledge of AB and their use	0.34	0.28	0.30, 0.39	<0.001
Knowledge of AB resistance	0.45	0.27	0.39, 0.51	<0.001

CI: Confidence interval; AB: antibiotics.

**Table 5:** Association between LA storage and disposal methods and the independent variables (n = 3191).

	Storage			Disposal		
	Correct n = 1758	Incorrect n = 1433		Correct n = 699	Incorrect n = 2492	
	n (%)	n (%)	*p*-Value	n (%)	n (%)	*p*-Value
Country			<0.001			<0.001
Egypt	878 (58.3)	627 (41.7)		302 (20.1)	1203 (79.9)	
Lebanon	285 (58.4)	203 (41.6)		84 (17.2)	404 (82.8)	
Jordan	226 (53.7)	195 (46.3)		30 (7.1)	391 (92.9)	
Serbia	127 (52.9)	113 (47.1)		146 (60.8)	94 (39.2)	
Bangladesh	85 (36.0)	151 (64.0)		63 (26.7)	173 (73.3)	
Brazil	88 (48.4)	94 (51.6)		50 (27.5)	132 (72.5)	
India	69 (58.0)	50 (42.0)		24 (20.2)	95 (79.8)	
Gender			0.031			0.218
Female	1111 (56.6)	852 (43.4)		416 (21.2)	1547 (78.8)	
Male	647 (52.7)	581 (47.3)		283 (23.0)	945 (77.0)	
Marital status			0.541			0.595
Single	1125 (55.5)	902 (44.5)		450 (22.2)	1577 (77.8)	
Ever married	633 (54.4)	531 (45.6)		249 (21.4)	915 (78.6)	
Education level			0.011			0.721
Low/none	23 (36.5)	40 (63.5)		15 (23.8)	48 (76.2)	
Moderate	278 (54.8)	229 (45.2)		117 (23.1)	390 (76.9)	
High	1457 (55.6)	1164 (44.4)		567 (21.6)	2054 (78.4)	
Crowding index			0.74			<0.001
No crowding	563 (55.5)	451 (44.5)		310 (26.1)	880 (73.9)	
Crowding	1195 (54.9)	982 (45.1)		389 (19.4)	1612 (80.6)	
Healthcare provider			0.39			<0.001
Yes	644 (54.1)	546 (45.9)		291 (28.7)	723 (71.3)	
No	1114 (55.7)	887 (44.3)		408 (18.7)	1769 (81.3)	
	Mean ± SD	Mean ± SD	*p*-value	Mean ± SD	Mean ± SD	*p*-value
Age	29.5 ± 11.02	29.1 ± 10.2	0.277	29.2 ± 13.7	29.7 ± 11.6	0.278
Knowledge about AB and AB use	53.5 ± 8.4	55.4 ± 8.4	<0.001	56.4 ± 8.9	53.8 ± 8.2	<0.001
Knowledge about AB resistance	43.3 ± 6.1	43.4 ± 6.1	0.490	45.2 ± 6.3	42.8 ± 5.9	<0.001

AB: antibiotics.

**Table 6:** Binary logistic regression analysis of factors associated with antibiotics storage and disposal practices (n = 3191).

	Correct Practice Regarding Antibiotic Storage			Correct Practice Regarding Antibiotic Disposal		
	OR	95% CI	*p*-Value	OR	95% CI ^1^	*p*-Value
Country Egypt (reference)						
Lebanon	1.12	0.76–1.64	0.56	1.16	0.72–1.86	0.55
Jordan	1.11	0.73–1.67	0.62	1.39	0.83–2.33	0.21
Serbia	0.87	0.57–1.32	0.5	3.19	1.77–5.75	<0.001
Bangladesh	1.03	0.65–1.63	0.9	0.18	0.11–0.31	<0.001
Brazil	0.46	0.29–0.74	0.001	0.63	0.37–1.09	0.1
India	0.79	0.49–1.27	0.33	0.78	0.44–1.37	0.39
Gender Female (reference)						
Male	1.16	1.00–1.35	0.05			
Educational level Low or none (reference)						
Moderate education	0.59	0.34–1.01	0.04			
Higher education	1.03	0.84–1.27	0.99			
Crowding index No (reference)				0.87	0.72–1.06	0.16
Yes						
Healthcare provider No (reference)				1.85	1.52–2.26	<0.001
Yes						
Knowledge of AB and AB use	0.96	0.95–0.97	<0.001	1.00	0.99–1.01	0.84
Knowledge of AB resistance	1.02	1.00–1.03	0.025	0.97	0.95–0.98	<0.001

^1^ 95% confidence interval for the odds ratio.

## Data Availability

Data is available upon request. Contact: katia_iskandar@hotmail.com.

## Abbreviations

The following abbreviations are used in this manuscript:

AB	Antibiotics
LAs	Leftover antibiotics
SMLA	Self-medication with leftover antibiotics
SD	Standard deviation
HICI	Household crowding index
KAP	Knowledge, attitude, and practice
LMICs	Low- and middle-income countries
HICs	High-income countries
WHO	World Health Organization

## Appendix A

### Appendix A.1. Knowledge of Antibiotics and Antibiotics Use

This section includes 15 statements, each rated on a 5-point Likert scale ranging from “strongly disagree” to “strongly agree”. Of these, 10 represent positive statements and 5 are negative statements. Positive statements are coded as follows: 1 for “Strongly disagree” 2 for “Disagree”, 3 for “Neither agree or disagree” 4 for “Agree”, and 5 for “Strongly agree”. Negative statements are reverse coded accordingly.

The statements of this scale are as follows:

- Antibiotics can cause allergic reactions.
- Antibiotics can cause vomiting and nausea.
- Antibiotics can cause diarrhea.
- Antibiotics can cause abdominal pain.
- Antibiotics can cause skin reactions (rashes/ulcers).
- Antibiotics can cause liver toxicity.
- Antibiotics can cause kidney toxicity.
- If you have side effects during a course of antibiotics treatment, you should see your doctor immediately.
- If you have a history of side effects or allergy to antibiotics, you should inform your doctor/pharmacist.
- Antibiotics can cause imbalance in the body’s own bacterial flora (good bacteria).
- It is okay to use leftover antibiotics from previous treatments.
- It is okay to use antibiotics that were given to a friend or family member, as long as they are used to treat the same illness.
- It is okay to save antibiotics for later use.
- It is okay to buy antibiotics without a prescription.
- I should stop taking antibiotics used for my treatment when I feel better.

### Appendix A.2. Knowledge About Antibiotic Resistance

This section includes 12 statements, each rated on a 5-point Likert scale ranging from “strongly disagree” to “strongly agree”. Of these, 10 represent positive statements and two represent negative statements. Positive statements are coded as follows: 1 for “Strongly disagree”, 2 for “Disagree”, 3 for “Neither agree or disagree” 4 for “Agree”, and 5 for “Strongly agree”. Negative statements are reverse coded accordingly.

The statements of this scale are as follows:

- Many infections are becoming increasingly resistant to treatment by antibiotics.
- Antibiotic resistance is an issue that could affect me and the future generation.
- Antibiotic resistance is an issue in other countries but not in my country.
- Antibiotic resistance is only a problem for people who take antibiotics regularly.
- Bacteria which are resistant to antibiotics can be spread from person to person.
- You can be a carrier of resistant bacteria and pass them to your friends and/or family members.
- Antibiotic-resistant infections could make medical procedures like surgery, organ transplants, and cancer treatment much more dangerous.
- If you become sick and your bacteria are resistant to your prescribed antibiotic, your illness could last longer.
- Taking antibiotics unnecessarily may contribute to the development of antibiotic resistance.
- Completing the course of antibiotic prescribed is important to completely kill the bacteria that cause your illness.
- The use of antibiotics among animals can reduce the effect of antibiotics among humans.
- Animals can act as carriers of resistant bacteria and pass them to human.

### Appendix A.3. Knowledge About the Risks of SMLA

This section includes 15 statements, each rated on a 5-point Likert scale ranging from “strongly disagree” to “strongly agree”. These statements are coded as follows: 1 for “Strongly disagree”, 2 for “Disagree”, 3 for “Neither agree or disagree”, 4 for “Agree”, and 5 for “Strongly agree”.

The statements of this scale are as follows:

- Incorrect self-diagnosis.
- Failure to seek appropriate medical advice promptly.
- Incorrect choice of therapy.
- Failure to recognize special pharmacological risks.
- Rare but severe adverse effects.
- Failure to recognize or self-diagnosis contraindications, interactions, warnings, and precautions.
- Failure to recognize that the same active substance is already being taken under a different name.
- Failure to report current self-medication to the prescribing physician (double medication/harmful interaction).
- Failure to recognize or report adverse drug reactions.
- Incorrect route of administration.
- Inadequate or excessive dosage.
- Excessively prolonged use.
- Risk of dependence and abuse.
- Food and drug interaction.
- Storage in incorrect conditions or beyond the recommended shelf life.

**Table A1:** Cumulative variance contribution and load factor.

Total Variance Explained						
Component	Initial Eigenvalues			Extraction Sums of Squared Loadings		
	Total	% of Variance	Cumulative %	Total	% of Variance	Cumulative %
1	8.630	57.531	57.531	8.630	57.531	57.531
2	0.954	6.361	63.892			
3	0.614	4.095	67.986			
4	0.580	3.867	71.854			
5	0.534	3.561	75.415			
6	0.490	3.266	78.681			
7	0.428	2.855	81.536			
8	0.417	2.778	84.314			
9	0.398	2.650	86.965			
10	0.379	2.528	89.493			
11	0.359	2.394	91.887			
12	0.336	2.240	94.127			
13	0.316	2.109	96.235			
14	0.294	1.963	98.198			
15	0.270	1.802	100.000			

Extraction Method: Principal Component Analysis.

**Table A2:** The component matrix.

Component Matrix	
	Component
	1
Misdiagnosis	0.643
Delayed consultation	0.733
Unaware of risks	0.783
Wrong therapy	0.773
Severe adverse effect	0.728
Contradiction ignorance	0.778
Duplicate medication	0.769
Unreported medication	0.770
Unreported adverse effect	0.793
Incorrect administration	0.763
Dosage error	0.813
Prolonged use	0.777
Drug misuse	0.764
Interaction risks	0.724
Improper storage	0.753
